# Prevalence of Vitamin D Deficiency in Children with Fractures: Before and during the COVID-19 Outbreak

**DOI:** 10.1155/2022/4410032

**Published:** 2022-06-22

**Authors:** Yong-Suk Lee, Sang-Uk Lee, Tae Min Hong, Sun Young Joo

**Affiliations:** Department of Orthopedic Surgery, Incheon St. Mary's Hospital, College of Medicine, The Catholic University of Korea, Seoul, Republic of Korea

## Abstract

**Background:**

Although it is generally agreed that vitamin D is important for bone health, the role of vitamin D in preventing fractures in children and adolescents remains unclear. Therefore, this study aimed to investigate the prevalence of vitamin D deficiency and insufficiency in healthy Korean children with fractures. Our secondary aim was to compare serum vitamin D levels before and during the coronavirus disease 2019 (COVID-19) outbreak.

**Methods:**

We evaluated 334 patients with fractures who were surgically treated at our institution between 2018 and 2019 before the onset of COVID-19 (group I). In addition, we collected data on the serum 25(OH)D levels of 210 patients who visited our pediatric department for evaluation of short stature (group II) and the serum 25(OH)D levels of the patients with fractures during the COVID-19 pandemic period (group III). A serum 25(OH)D level of <20 ng/mL was considered deficient, between 20 and 32 ng/mL was insufficient, and ≥32 ng/mL was considered sufficient.

**Results:**

The mean age was 8.1 ± 3.5 years in group I, 8.2 ± 3.7 years in group II, and 8.6 ± 3.5 years in group III. The prevalence of vitamin D deficiency was 53.0% in group I and 32.9% in group II. The mean serum 25(OH)D level was lower in group I than in group II (20.0 ± 7.3 ng/ml vs. 23.2 ± 6.9 ng/ml, *p* < 0.001). The mean serum 25(OH)D level of younger patients (<10 years) in group III was lower than that of the younger patients in the prepandemic period (21.4 ± 7.2 ng/mL vs. 19.2 ± 6.8 ng/mL, *p*=0.037).

**Conclusions:**

We observed a high prevalence of vitamin D deficiency/insufficiency in children with fractures who required surgical treatment. During the COVID-19 pandemic, the serum vitamin D levels became even lower, especially in younger children.

## 1. Introduction

Vitamin D is a fat-soluble vitamin that promotes the efficient absorption of dietary calcium and phosphorus and is therefore essential for bone growth and mineralization [1]. Vitamin D is synthesized in the skin during exposure to ultraviolet-B radiation from sunlight, and only small amounts can be obtained (<10%) from dietary intake [1]. Skeletal complications of vitamin D deficiency include rickets, bone deformities, osteoporosis, and fractures. Vitamin D deficiency has also been associated with cardiovascular disease, diabetes mellitus, cancers, infections, autoimmune diseases, and schizophrenia [2]. Due to a deeper understanding of its influence on overall health, interest in the role played by vitamin D in bone health is increasing.

The relationship between low vitamin D levels and fracture risk has been studied extensively, and there is ample evidence that vitamin D insufficiency is associated with an increased risk of osteoporotic fracture in adults [3, 4]. It is also well known that children with radiographically confirmed rickets have an increased risk of bone fracture [5, 6]. Chapman et al. reported that in 45 patients less than two years old with rickets, fractures were present in 17.5% [6]. Although it is generally agreed that vitamin D is important for bone mineralization, the role of vitamin D in preventing fractures among children and adolescents remains unclear.

In addition, daily life has drastically changed since the World Health Organization declared the coronavirus disease 2019 (COVID-19) pandemic on March 11, 2020 [7]. Many countries have implemented policies to restrict daily life, such as social distancing and lockdowns, to reduce the spread of COVID-19. Home confinement resulted in a reduction in children's activities such as attending school, organized sports, and playground use. As a result, pediatric fracture volume has decreased during the COVID-19 pandemic [8, 9]. Several studies have looked at the effect of COVID-19 on children's physical activity and obesity; however, no study has examined the vitamin D status in children with fractures during the COVID-19 outbreak [10–12]. Theoretically, we would expect home confinement and limited outdoor activity to result in decreased sunlight exposure, thus decreasing serum vitamin D.

Therefore, this study primarily aimed to investigate the prevalence of vitamin D deficiency and insufficiency in healthy Korean children with sustained fractures. The second aim of this study was to compare serum vitamin D levels before and during the COVID-19 pandemic. We hypothesized that there would be a high prevalence of vitamin D deficiency in children with fractures during the COVID-19 pandemic period due to limited sun exposure from reduced outdoor activities.

## 2. Materials and Methods

We conducted a retrospective study after obtaining approval from our institutional review board. From electronic medical records, we evaluated 334 pediatric patients with fractures who were surgically treated at our institution between 2018 and 2019 (prepandemic period, group I). Patients were excluded based on the presence of a pathologic fracture, metabolic disorder, neuromuscular disease, or history of medication that may have affected bone health. The control group consisted of children who visited our pediatric department for evaluation of short stature during the same period. We excluded patients if they had any endocrinologic abnormalities. In total, 210 patients met the control group criteria (group II). Finally, to compare the prevalence of vitamin D deficiency before and during COVID-19, a third group comprised 97 patients surgically treated for fractures from January 2020 to December 2020 (group III). Patient demographics such as age, sex, site of injury, age at presentation, and body mass index (BMI) were collected from the medical records.

We measured the serum 25(OH)D level of the patients, known to be the best indicator of vitamin D status. A serum 25(OH) D level of <20 ng/mL was considered deficient, a level between 20 and 32 ng/mL was insufficient, and≥32 ng/mL was considered sufficient based on the previously published literature [13].

The date of presentation was categorized into four seasons: spring (March to May), summer (June to August), autumn (September to November), and winter (December to February). The fracture site was categorized as upper extremity, lower extremity, and fracture of an appendage (hand or foot). Age was grouped as younger children, with children who were less than 10 years old and the adolescent group (≥10 years). The BMI was calculated from body weight and height. A BMI that was less than the 85th percentile was considered normal and above the 85th percentile was considered overweight.

A normality test was performed using the Kolmogorov–Smirnov test before statistical analysis. Student's *t*-test was used to analyze the differences in continuous variables, and the chi-square test or Fisher exact test was used to investigate differences in distribution among groups. Statistical analyses were performed using SPSS software version 21 (IBM Co., Armonk, NY, USA). Statistical significance was set at *p* < 0.05.

## 3. Results

The demographic and clinical characteristics of the participants are summarized in Table 1. There were more boys than girls in all groups: 229 boys (68.6%) and 105 girls (31.4%) in group I, 118 boys (56.2%) and 92 girls (43.8%) in group II, and 54 boys (55.7%) and 43 girls (44.3%) in group III. The mean age in group I was 8.2 ± 3.7 years, 8.5 ± 3.7 years in the control group, and 8.6 ± 3.5 years in group III. The mean BMI was slightly higher in group III compared to group I; however, it was not statistically significant (19.2 ± 3.9 kg/m^2^ vs. 18.8 ± 4.3 kg/m^2^, *p*=0.574). The mean serum 25(OH)D level was significantly lower in group I compared to group II (21.4 ng/mL vs. 23.20 ng/mL, *p*=0.020).

Table 2 summarizes the site of injury in patients. The incidence of fracture decreased from 334 patients during 2018–2019 to 97 patients during COVID-19 (2020). Upper extremity fractures were more common than lower extremity fractures in both groups, with the supracondylar as the most common fracture site observed in 79 patients (23.7%) before COVID-19 and 23 patients (23.7%) during COVID-19.

Table 3 shows the prevalence of vitamin D deficiency or insufficiency in each group. The overall prevalence of vitamin D deficiency was 53.0% in group I, 32.9% in group II, and 66.0% during the COVID-19 period. The serum vitamin D level was sufficient in only 5.1% in group I, 11.0% in group II, and 4.1% in group III. There was significant difference in the distribution of sufficient, insufficient, and deficient between group I and group II (*p* < 0.001). However, there was no difference in the distribution between group I and group III (*p*=0.075).

Table 4 presents the mean serum 25(OH)D levels and prevalence of vitamin D deficiency according to the season. The mean serum 25(OH)D level was lower in winter and spring and higher in summer and autumn, regardless of the group. Likewise, the prevalence of vitamin D deficiency was the highest in winter in groups I and III (69.5% and 93.3%, respectively), and in group II, it was the highest in spring (57.4%). Compared to group II, the mean serum 25(OH)D level was significantly lower in group I regardless of the season. The distribution of sufficient, insufficient, and deficient between group I and group II was different in summer, autumn, and winter (*p* < 0.001, *p* < 0.001, and *p*=0.009, respectively). Compared to group I, the mean serum 25(OH)D level tended to be lower in group III; however, the difference was not statistically significant.

Table 5 compares the serum 25(OH)D levels of each group according to age group, sex, and BMI. The 25(OH)D levels were higher in younger children than in adolescents and higher in normal-weight children than in obese children. Comparing the mean serum 25(OH)D levels between groups I and II, the level was significantly lower in group I, regardless of age group, sex, or BMI. The mean serum 25(OH)D level in younger patients in group III was significantly lower than that in group I (19.2 ± 6.8 vs. 21.4 ± 7.2. *p*=0.037).

## 4. Discussion

This study aimed to investigate the prevalence of vitamin D deficiency and insufficiency in healthy Korean children with sustained fractures as well as to compare serum vitamin D levels before and during the COVID-19 outbreak. Our results show a high prevalence of vitamin D deficiency and insufficiency in children with fractures and the control group (94.9% and 89.1%, respectively). A high prevalence of hypovitaminosis D was observed during the COVID-19 outbreak, especially in younger patients compared to older children.

In this study, we selected a cutoff value for serum vitamin D to define deficiency, but the optimal level is controversial. For example, the Institute of Medicine places the optimal level at 20 ng/mL, whereas the Endocrine Society defines it as > 30 ng/mL [14, 15]. The Endocrine Society is the most frequently used definition with deficiency defined as < 20 ng/mL, insufficiency as ≤ 30 ng/mL, and sufficiency as > 30 ng/mL [16]. However, we defined deficiency as < 20 ng/mL since there is evidence to suggest that there is a significant difference in bone quality in individuals with a serum vitamin D level of 20 vs. 30 ng/mL. [16] Furthermore, a recent comparative cohort study from Fabricant et al. showed that pediatric patients with low-energy fractures had low serum 25(OH)D levels comparable to individuals with chronic kidney disease (27.5 ng/mL and 24.6 ng/mL, respectively). [16] Therefore, they suggest using a cutoff of 32 ng/mL for vitamin D insufficiency as more appropriate for children.

Korean children are at an increased risk for vitamin D deficiency because of the high latitude (34–38°N), seasonal effects, increased use of sunscreen, reduced outdoor activity, and lack of vitamin D-fortified foods. The prevalence of vitamin D deficiency in healthy Korean children and adolescents has not been well studied. Yoon et al. measured the serum vitamin D levels in 171 Korean children aged <2 years and found that the prevalence of vitamin D insufficiency (serum level <30 ng/mL) was 29.8% [17]. Kim et al. evaluated the prevalence and risk factors of vitamin D deficiency in 2062 Korean adolescents and reported a mean serum vitamin D level of 17.68 ng/mL. In addition, 13.4% patients had serum 25(OH)D levels <10 ng/mL, while 68.1% had levels <20 ng/mL [18]. Unfortunately, there is a lack of information on the prevalence of vitamin D deficiency in Korean children aged 2–10 years. In our study, the prevalence of vitamin D deficiency in the healthy control group was 32.9%. This is higher than reported in the study by Yoon et al., which included younger children aged <2 years and lower than the study by Kim et al., which included only adolescents. We believe that this is because serum 25(OH)D levels decrease significantly with increasing age, as suggested by previous studies. [19] Regardless of the age group, the serum 25(OH)D levels in the present study were lower in winter and spring and higher in summer and autumn, consistent with the findings from other studies [20].

The association between mild vitamin D deficiency and fracture risk in healthy children remains debatable. A previous global consensus statement indicated that children with radiographically confirmed rickets have an increased risk of fracture and that children with mild vitamin D deficiency do not have an increased risk of fracture based on observational studies and case reports [21]. Ryan et al. evaluated vitamin D status and the risk of forearm fracture [22]. Their study included 76 patients and 74 controls with an age range of 5 to 9 years. Compared with the control group, the patient group showed lower bone mineral density and was more likely to be vitamin D deficient (47.1% vs. 40.8%). Likewise, Saglam et al. compared the vitamin D levels of 30 fracture patients with 30 controls and found that vitamin D levels were significantly lower in patients with forearm fractures (14.5 ng/mL vs. 21.3 ng/mL, *p*=0.002) [23]. In contrast, Minkowitz et al. failed to show a significant difference between vitamin D levels and the risk of fracture in a study that compared 369 fracture patients with 662 nonfracture controls aged 18 years and younger and find no differences in vitamin D status (27.5 ± 8.9 ng/mL vs. 27.4 ± 9.1 ng/mL) [24]. However, in the same study, children with lower vitamin D levels were at a higher risk of developing more severe fractures. In addition, a recent systematic review and meta-analysis that included 17 case-control and 6 cross-sectional studies (2929 fracture cases and 5000 controls) demonstrated that serum 25(OH)*D* ≤ 50 nmol/L (or 20 ng/mL) was associated with increased fracture risk in children [25]. In the present study, our patient group showed a high prevalence of vitamin D deficiency (<20 ng/mL) in children with fractures compared to the controls (53.0% vs. 32.9%). The prevalence was even higher during the COVID-19 pandemic (66.0%).

Since the COVID-19 outbreak, studies have been conducted to evaluate its effect resulting from social distancing/lockdown measures on pediatric bone health and physical activity. Although social distancing is critical in reducing the transmission of COVID-19, it may also have adverse effects on children's health. School closures were mandated by many governments. However, aside from the hindrance in learning and resulting disparities in education, the physical activity of children was drastically reduced, and there are a few studies on the association between the COVID-19 outbreak and the fracture incidence in children [8–10, 26, 27]. Dunton et al. reported decreased physical activity and increased sedentary behavior during the early stages of COVID-19 presumably due to the closure of schools and parks and the cancellation of youth sports and other activities [10]. The pediatric fracture volume has decreased during the COVID-19 pandemic, partially, because of the cessation of organized sports and decreased playground use. In China, home confinement led to a significant reduction in the number of pediatric fractures, which decreased from 5346 patients in 2017–2019 to 862 patients in 2020 [26]. A similar finding was observed in the Korean population. The number of patients visiting the emergency department in 2021 decreased by 43% compared to that in the last three years [27]. Similarly, in the present study, the number of patients with fractures was reduced from 334 patients in 2018–2019 to 97 patients in 2020.

Weight gain in children during the COVID-19 lockdown has also been suggested as an adverse effect of social distancing according to studies from Israel, Korea, and Italy where the risk of childhood obesity has significantly increased because of a lack of activity [11, 12, 28]. In Israel, during 7 weeks of lockdown, general weight gain was observed (increasing from the 38.82 percentile to the 40.44 weight percentile) [28]. Similar findings were observed in Korea, where BMI z-scores increased by 0.219 (95% confidence interval (CI), 0.167–0.271; *p* < 0.001) when compared to the pre-COVID-19 period in 226 children between 4 and 14 years of age during COVID-19, and the proportion of overweight or obesity increased from 23.9% in the pre-COVID-19 period to 31.4% in the COVID-19 period [11]. Although it was not significantly different in our patients, the mean BMI tended to be higher in the COVID-19 period than the prepandemic period (19.2 ± 3.9 kg/m^2^ vs. 18.8 ± 4.3 kg/m^2^, *p*=0.574).

To the best of our knowledge, this is the first study to evaluate vitamin D status in children with fractures during the COVID-19 outbreak. Compared to the serum level of 25(OH)D in the patient group before the COVID-19 outbreak, the overall serum level tended to be lower in the COVID-19 cohort but without a significant difference (20.0 ± 7.3 ng/mL vs. 18.8 ± 7.3 ng/mL, *p*=0.056). However, in the younger patients aged less than 10 years, there was a significantly lower serum level of 25(OH)D during COVID-19 compared to before COVID-19 (21.4 ± 7.2 ng/mL vs. 19.2 ± 6.8 ng/mL, *p*=0.037). We propose younger patients were more affected by the lack of outdoor activity as a result of social distancing. As the outbreak accelerated, the Korea Disease Control and Prevention Agency, together with the Ministry of Education, inevitably decided to delay school opening and switched to online learning for eight weeks. After that, even with the partial opening of the schools, playgrounds and public parks were closed, and organized sports activities were prohibited.

The strength of this study is that there has been no previous study that examines serum vitamin D levels in children with fractures during COVID-19. However, our study has some limitations. First, due to the retrospective nature of this study, we were not able to include all variables, such as sunlight exposure and diet intake, which may have affected the results. Second, secondary hyperparathyroidism resulting from vitamin D deficiency has been associated with impaired bone microarchitecture according to the literature [29]. However, the serum parathyroid level results were not available for use in our study. Third, the causes of fractures are multifactorial. In addition to the strength of the bone, muscle imbalance or the amount of force can contribute to their cause. Finally, we included only surgically treated patients. Patients admitted for fractures generally had severe fractures that required intensive treatment; therefore, this study did not include those milder fractures that did not require surgical treatment.

## 5. Conclusion

Vitamin D deficiency/insufficiency was very common in our pediatric population with fractures. During the COVID-19 pandemic, serum 25(OH)D levels were lower than before the pandemic. Decreased outdoor activity due to social distancing and school closures may have contributed to lower serum vitamin D levels, especially in younger children. Further studies are needed to identify children at increased risk of fracture and determine optimal serum vitamin D levels to promote fracture healing.

## Figures and Tables

**Table 1:** Patient demographics.

	Group I (*N* = 334)	Group II (*N* = 210)	Group III (*N* = 97)	*p* value^*∗*^	*p* value^†^
Age (years)	8.2 ± 3.7	8.5 ± 3.7	8.6 ± 3.5	0.409	0.357
M : F	229 (68.6%):105 (31.4%)	118 (56.2%):92 (43.8%)	54 (55.7%):43 (44.3%)	0.003	0.019
BMI (kg/m^2^)	18.8 ± 4.3	18.0 ± 3.1	19.2 ± 3.9	0.147	0.574
Season				<0.001^#^	0.045^#^
Spring	62 (18.6%)	54 (25.7%)	31 (32.0%)		
Summer	118 (35.3%)	64 (30.5%)	29 (29.9%)		
Autumn	95 (28.4%)	32 (15.2%)	22 (22.7%)		
Winter	59 (17.7%)	60 (28.6%)	15 (15.5%)		
25(OH)D (ng/mL)	20.0 ± 7.3	23.2 ± 6.9	18.8 ± 7.3	<0.001	0.056

Data are presented as the mean ± standard deviation or *n* (%). BMI, body mass index. ^*∗*^*p* value: compared group I with group II, ^†^*p* value: compared group I with group III, and ^*#*^*p* value: calculated by the chi-square test.

**Table 2:** Details of the fractures.

Location	Group I			Group III		
	N (%)	Age (years)	25(OH)D	N (%)	Age (years)	25(OH)D
			(ng/ml)			(ng/ml)
Upper extremity	237 (71.0%)			69 (71.1%)		
Humerus shaft	9 (2.7%)	10.9 ± 2.7	18.3 ± 4.7	4 (4.1%)	11.8 ± 3.9	16.4 ± 3.5
Supracondyle	79 (23.7%)	6.3 ± 2.6	20.8 ± 8.0	23 (23.7%)	6.4 ± 1.8	18.7 ± 6.4
Lateral condyle	53 (15.9%)	5.0 ± 1.9	21.4 ± 6.8	15 (15.5%)	5.4 ± 1.6	19.0 ± 6.1
Medial epicondyle	16 (4.8%)	9.3 ± 2.7	16.5 ± 5.9	5 (5.2%)	10.4 ± 4.2	16.8 ± 2.2
Forearm						
Proximal	31 (9.3%)	7.3 ± 3.1	21.2 ± 7.0	9 (9.3%)	8.3 ± 1.7	20.3 ± 4.1
Shaft	25 (7.5%)	8.8 ± 3.2	21.0 ± 7.8	2 (2.1%)	10.0 ± 1.4	18.7 ± 3.8
Distal	24 (7.2%)	9.2 ± 3.7	21.8 ± 7.0	11 (11.3%)	10.1 ± 3.3	19.4 ± 9.4
Lower extremity	53 (15.9%)			20 (20.6%)		
Femur	13 (3.9%)	8.9 ± 3.9	18.4 ± 8.7	4 (4.1%)	6.5 ± 4.8	17.7 ± 0.6
knee	7 (2.1%)	11.4 ± 3.4	16.6 ± 6.3	2 (2.1%)	12.0 ± 1.4	10.8 ± 4.8
Tibia	4 (1.2%)	9.3 ± 4.8	23.1 ± 4.5	4 (4.1%)	13.0 ± 2.0	15.6 ± 5.5
Ankle	29 (8.7%)	11.6 ± 3.1	15.3 ± 7.0	10 (10.3%)	11.6 ± 1.4	17.4 ± 5.1
Hand and foot	44 (13.2%)	11.5 ± 3.0	21.1 ± 4.4	8 (8.2%)	10.6 ± 4.5	20.6 ± 11.3
Total	334 (100%)	8.2 ± 3.7	20.0 ± 7.3	97 (100%)	8.6 ± 3.5	18.5 ± 6.5

Data are presented as the mean ± standard deviation, or *n* (%).

**Table 3:** Prevalence of vitamin D deficiency of the patients.

Variable	Group I (*N* = 334)	Group II (*N* = 210)	Group III (*N* = 97)	*p* value^*∗*^	*p* value^†^
Serum 25(OH)D				<0.001^#^	0.075^#^
Deficient	177 (53.0%)	69 (32.9%)	64 (66.0%)		
Insufficient	140 (41.9%)	118 (56.2%)	29 (29.9%)		
Sufficient	17 (5.1%)	23 (11.0%)	4 (4.1%)		

Data are presented as *n* (%). Deficient: serum 25(OH)D level <20 ng/mL, insufficient: serum 25(OH)D level from 20 to 32 ng/mL, and sufficient: serum 25(OH)D level ≥32 ng/mL. ^*∗*^*p* value: compared group I with group II, ^†^*p* value: compared group I with group III, and ^*#*^*p* value: calculated by the chi-square test.

**Table 4:** The serum 25(OH)D level of the patients according to season.

	Group I (*N* = 334)	Group II (*N* = 210)	*p* value^∗^	Group III (*N* = 97)	*p* value^†^
*Spring*			0.392^#^		0.150^#^
Deficient	41 (66.1%)	31 (57.4%)		26 (83.9%)	
Insufficient	18 (29.0%)	17 (31.5%)		5 (16.1%)	
Sufficient	3 (4.8%)	6 (11.1%)		0 (0%)	
Serum vit D	18.8 ± 7.7	21.3 ± 7.4	0.049	16.8 ± 4.1	0.161
*Summer*			<0.001^#^		0.915^#^
Deficient	49 (41.5%)	9 (14.1%)		13 (44.8%)	
Insufficient	62 (52.5%)	46 (71.9%)		14 (48.3%)	
Sufficient	7 (5.9%)	9 (14.1%)		2 (6.9%)	
Serum vit D	22.1 ± 6.6	25.5 ± 5.7	0.001	21.1 ± 7.3	0.474
*Autumn*			<0.001^#^		0.755^#^
Deficient	46 (48.4%)	4 (12.5%)		11 (50.0%)	
Insufficient	44 (46.3%)	25 (78.1%)		9 (40.9%)	
Sufficient	5 (5.3%)	3 (9.4%)		2 (9.1%)	
Serum vit D	20.2 ± 6.7	24.6 ± 6.4	0.001	19.7 ± 7.6	0.774
*Winter*			0.009^#^		0.165^#^
Deficient	41 (69.5%)	25 (41.7%)		14 (93.3%)	
Insufficient	16 (27.1%)	30 (50.0%)		1 (6.7%)	
Sufficient	2 (3.4%)	5 (8.3%)		0 (0%)	
Serum vit D	17.5 ± 8.0	21.7 ± 7.2	0.001	15.1 ± 5.0	0.287

Data are presented as mean ± standard deviation, or *n* (%). Deficient: serum 25(OH)D level <20 ng/mL, insufficient: serum 25(OH)D level from 20 to 32 ng/mL, and sufficient: serum 25(OH)D level ≥32 ng/mL. ^*∗*^*p* value: compared group I with group II, ^†^*p* value: compared group I with group III, and ^*#*^*p* value: calculated by the chi-square test.

**Table 5:** Serum 25(OH)D levels according to age group, sex, and BMI.

	Group I (*N* = 334)	Group II (*N* = 210)	Group III (*N* = 97)	*p* value^*∗*^	*p* value^†^
Age (years)					
<10	21.4 ± 7.2	24.7 ± 7.1	19.2 ± 6.8	<0.001	0.037
≥10	17.6 ± 7.0	20.2 ± 5.5	17.2 ± 5.8	0.008	0.766
Sex					
Boys	20.2 ± 7.5	23.6 ± 6.7	18.2 ± 6.6	<0.001	0.071
Girls	19.5 ± 7.0	22.7 ± 7.2	18.8 ± 6.5	0.002	0.605
BMI (kg/m^2^)					
Normal	21.2 ± 7.4	23.4 ± 6.9	19.4 ± 7.5	0.011	0.174
Overweight	18.3 ± 7.0	22.2 ± 6.3	16.7 ± 5.7	<0.001	0.232

Data are presented as mean ± standard deviation or *n* (%). BMI, body mass index. ^*∗*^*p* value: compared group I with group II and ^†^*p* value: compared group I with group III.

## Data Availability

Data that support the findings of this study are available from the corresponding author on request. Data are not publicly available due to privacy or ethical restrictions.
